# Microorganisms as bio‐filters to mitigate greenhouse gas emissions from high‐altitude permafrost revealed by nanopore‐based metagenomics

**DOI:** 10.1002/imt2.24

**Published:** 2022-05-08

**Authors:** Chenyuan Dang, Ziqi Wu, Miao Zhang, Xiang Li, Yuqin Sun, Ren'an Wu, Yan Zheng, Yu Xia

**Affiliations:** ^1^ School of Environmental Science and Engineering, College of Engineering Southern University of Science and Technology Shenzhen China; ^2^ Laboratory of High‐Resolution Mass Spectrometry Technologies, Dalian Institute of Chemical Physics Chinese Academy of Sciences (CAS) Dalian China; ^3^ Shenzhen Key Laboratory of Marine Archaea Geo‐Omics, Department of Ocean Science and Engineering Southern University of Science and Technology Shenzhen China; ^4^ State Environmental Protection Key Laboratory of Integrated Surface Water‐Groundwater Pollution Control, School of Environmental Science and Engineering Southern University of Science and Technology Shenzhen China

**Keywords:** frame‐shift correction, global warming, high‐altitude permafrost, metatranscriptome, nanopore sequencing

## Abstract

The distinct climatic and geographical conditions make high‐altitude permafrost on the Tibetan Plateau suffer more severe degradation than polar permafrost. However, the microbial responses associated with greenhouse gas production in thawing permafrost remain obscured. Here we applied nanopore‐based long‐read metagenomics and high‐throughput RNA‐seq to explore microbial functional activities within the freeze‐thaw cycle in the active layers of permafrost at the Qilian Mountain. A bioinformatic framework was established to facilitate phylogenetic and functional annotation of the unassembled nanopore metagenome. By deploying this strategy, 42% more genera could be detected and 58% more genes were annotated to nitrogen and methane cycle. With the aid of such enlarged resolution, we observed vigorous aerobic methane oxidation by *Methylomonas*, which could serve as a bio‐filter to mitigate CH_4_ emissions from permafrost. Such filtering effect could be further consolidated by both on‐site gas phase measurement and incubation experiment that CO_2_ was the major form of carbon released from permafrost. Despite the increased transcriptional activities of aceticlastic methanogenesis pathways in the thawed permafrost active layer, CH_4_ generated during the thawing process could be effectively consumed by the microbiome. Additionally, the nitrogen metabolism in permafrost tends to be a closed cycle and active N_2_O consumption by the topsoil community was detected in the near‐surface gas phase. Our findings reveal that although the increased thawed state facilitated the heterotrophic nitrogen and methane metabolism, effective microbial methane oxidation in the active layer could serve as a bio‐filter to relieve the overall warming potentials of greenhouse gas emitted from thawed permafrost.

## Highlights

- On‐site MinION metagenomics and RNA‐seq were combined to explore microbial functionalities of active layers of the soil of Qilian Mountain permafrost.
- With the developed annotation pipeline‐FUNpore, 42% more genera and 58% more genes were detected.
- Active methane consumption by *Methylomonas* could serve as a biofilter to mitigate CH_4_ emission from permafrost.
- Dissimilatory nitrate reduction to ammonia pathway enabled a closed microbial nitrogen cycle and N_2_O consumption was observed.

## Graphical abstract

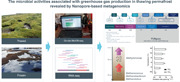

## INTRODUCTION

About 25% of the Earth's terrestrial surface is covered by permafrost, and it contains an estimated 1672 Pg carbon, which is roughly equal to the total carbon within vegetation and the atmosphere [1,2]. The overlaying permafrost soil is exposed to seasonal freeze‐thaw cycles and is called the active layer. Over the past few decades, the thickness of the active layer gradually increased, and the underlying permafrost declined or even disappeared in many places in the context of global warming [3–6]. With the thawing of permafrost, it can be predicted that an estimated 174 Pg of near‐surface (within 3 m of the surface) carbon will be accessible for microbial degradation by 2100 [7]. Owing to global warming, the substantial trapped carbon may be degraded by the revived microorganisms and result in greenhouse gas emissions [8,9].

In recent years, an increasing number of studies have focused on the microbial community, function, and activity involved in the microbial metabolism of organic carbon in permafrost [10–12]. Previous studies show that permafrost hosts a diversity of the microbial community, although it is exposed to harsh conditions [13,14]. These microorganisms can not only survive and grow but also perform metabolic functions [15,16], indicating that the microorganisms have adapted to the permafrost biophysical environment [17]. Recently, some studies show the shifts in the potential active bacterial communities between the thawing and freezing of an active layer of permafrost soil [18]. Additionally, with thawing, the microbial compositions and metabolic activities reveal rapid responses, especially those genes involved in carbon and nitrogen metabolism, which play a very important role in the degradation of organic matter [12,19,20]. Importantly, the three most important greenhouse gases: carbon dioxide (CO_2_), methane (CH_4_), and nitrous oxide (N_2_O) were all directly related to microbial carbon and nitrogen metabolism. Nevertheless, most of these studies about the permafrost microbial metabolism have been primarily conducted in polar regions.

The Qinghai‐Tibet Plateau is the largest cryosphere outside the Arctic and Antarctic [21] and is the largest high‐altitude permafrost region around the world [22]. Due to relatively young geological features and steep geothermal gradient, the permafrost on the Qinghai‐Tibet Plateau is thinner and warmer than permafrost in polar regions [23]. Moreover, the strong solar radiation [21], scarce organic layer [24], and regional stronger warming of the atmosphere [25] make the high‐altitude permafrost suffer a greater degree of degradation compared to high‐latitude permafrost in polar regions. The thawing of high‐altitude permafrost caused by the distinct climatic and geographical conditions is expected to affect the microbial communities and functions. However, although the microbial communities in high‐altitude permafrost in the Qinghai‐Tibet Plateau have been extensively investigated (mainly using an amplicon‐based approach) [22,26–28], the microbial functional composition and metabolic activity affected by global warming remain unclear in this region.

Here, we investigated the microbiome in the active layer of permafrost across three different altitudes (3000, 3500, and 4000 m) and compared microbial metabolic activities between thawed (sampling in August) and frozen (sampling in November) permafrost soils (3500 m), at the Qilian Mountain in the Qinghai‐Tibet Plateau, using the Oxford Nanopore Technologies (ONT)‐based metagenomic and Illumina‐based RNA‐seq, with the aim of (1) providing a new bioinformatic framework to analyze the functionality of the large unassembled proportion of soil metagenome with the aid of nanopore long‐read sequencing; (2) revealing the shift of microbial activities involved in greenhouse gas emission between thawed and frozen state of high‐altitude permafrost soil.

## RESULTS AND DISCUSSION

### Nanopore reads correction and functional prediction using FUNpore framework

**Figure 1:**
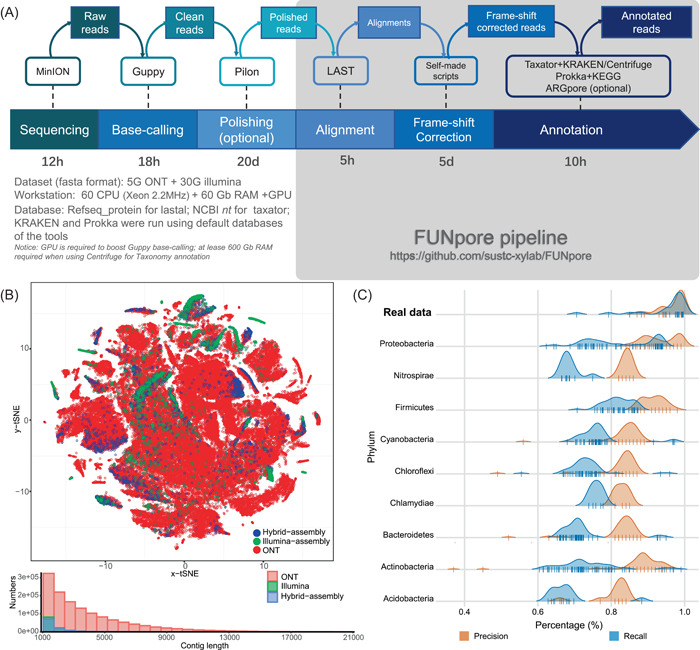
(A) The correction‐based annotation workflow of FUNpore. (B) The t‐distributed stochastic neighbor embedding (t‐SNE) analysis based on five‐nucleotide frequency of the nanopore reads, Illumina‐assembly (CLC genomic workbench 12.0), and hybrid‐assembly (OPERA‐MS) contigs. The histogram shows the distribution of length of nanopore reads, Illumina‐assembly, and hybrid‐assembly contigs. (C) Precision and recall of FUNpore functional annotation evaluated by the real nanopore sequencing data (highlighted upmost plot) and mock nanopore data set. The real data was the nanopore whole genome sequence of 20 isolated strains. The mock data was downloaded from the NCBI genome database and randomly introduced a 5% error base.

Although long nanopore reads can dramatically reduce the level of assembly fragmentation for genome recovery of isolated strains and ZymoBIOMICS mock community [29], for metagenomic data sets, neither assembly of nanopore reads alone nor a hybrid‐assembly of nanopore  reads and high‐quality Illumina short reads performed well in terms of reads usage ratio and assembly continuity (Figure 1B). A very low proportion of genomic information carried by the nanopore data set can be incorporated into the final assembly (Figure 1B), not to mention the dramatically inflated computational demand (mainly on server RAM) to include the nanopore data set for assembly. For our permafrost community, hybrid‐assembly of 5.2 Gbp nanopore data set with an average read length of 3.8 kb and 42 Gbp 150 bp paired‐end Illumina reads using popular hybrid assemblers of metaSPAdes and Opera‐MS barely improved the contig continuity compared to Illumina‐assembly (Figure 1B and Supporting Information: Table S5) and 67.4% of the nanopore data set were unused in the hybrid assembly (Supporting Information: Table S5). The current hybrid assemblers did not pay proper appreciation of the long noisy signal of nanopore long‐reads that the randomly distributed low‐quality regions on nanopore reads were virtually all cut open by the assembly algorithm because these regions could not be mapped by any of the Illumina short‐reads (Supporting Information: Figure S2). This resulted in the long reads being cut into fragments similar to the contigs assembled from short‐reads alone, losing the advantage of long‐reads in the hybrid assembly process. Moreover, assembly tools particularly designed for nanopore reads, such as Miniasm and Wtdbg2, also suffered from a high level of data loss for metagenomic data sets. Only around 5.3 and 28.0 Mbp genomic information was retrieved from the 5.2 Gbp permafrost nanopore data set (Supporting Information: Table S5). The major reason for this was the general lack of enough coverage caused by the high diversity within the metagenomic data set, which was unlikely to be addressed by algorithm improvement.

Therefore, to maintain as much as possible of the genomic information carried by nanopore data sets, the metagenomic analysis of this study used another increasingly popular approach: correction‐based annotation. First, bases with overly low quality (Qscore lower than 5) were replaced with N to avoid unexpected interference for subsequent correction. Next, SNP and Indel were corrected based on the alignment of massive NGS short reads against nanopore reads by Pilon [30]. The bwa with “mem” parameter showed the best performance based on the tradeoff between mapping sensitivity and run‐time among mainstream mapping tools, including bwa, bbmap, minimap2, and LAST (Supporting Information: Table S6). Based on our evaluation of a 15,000‐reads subset of the nanopore dates, the average base accuracy increased from 94% to 96% after Pilon polish (Supporting Information: Figure S3). However, there were only 59.01% nanopore reads had >50% bases polished, while 29.40% had >80% bases polished (Supporting Information: Figure S3), suggesting a large proportion of nanopore reads were unpolishable because of the unmappable low‐quality regions on nanopore reads as mentioned above (Supporting Information: Figure S2). Therefore, as the final step, we further corrected the distinguishable frame‐shifts caused by the remaining errors on nanopore reads based on LAST's frame‐shift‐aware DNA‐to‐protein alignment against the high‐quality NCBI Refseq_protein database. Inspired by the frame‐shift correction algorithm of MEGAN6 based on DIAMOND's frame‐shift‐aware alignment [31], we developed our own scripts for frame‐shifts correction based on the LAST alignment. Since the LAST alignment is much longer than DIAMOND's, more frame‐shift errors in nanopore reads could be corrected by FUNpore. We observed an average of 27.0 frameshifts per kilobyte of the aligned sequence by FUNpore (Supporting Information: Table S3), while 14.8 was reported based on DIAMOND alignment by MEGAN6 [31].

Functional prediction accuracy based on postcorrection nanopore reads was evaluated. Despite the large number of fake genes called by Prodigal, functional predictions based on the postcorrection nanopore data set showed high consistency (average precision of 96.6% and recall of 94.5%) to that of the golden standard, hybrid assembly (Figure 1C). The performance of the mock nanopore data set was a bit lower (precision varied from 80% to 94%, recall ranged from 65% to 97%), mainly owing to the fact that the distribution pattern of randomly introduced errors in the mimic data set was different to that of the real nanopore reads. Based on the mimic nanopore data set, visible variation among different phyla was observed, and the reads of *Proteobacteria*, *Firmicutes*, and *Actinobacteria* showed the highest accuracy of predicted functional categories (Figure 1C). Such high correction efficiency of these common phyla was presumably caused by their high representation in the Refseq_protein database, which could produce more alignments to facilitate FUNpore frame‐shift correction. To improve the usability of the FUNpore framework, we also included a phylogeny annotation step in the FUNpore framework by combing the results of Taxator and KRAKEN (Figure 1A).

With the aid of the assembly‐free long‐read metagenomic approach, we were able to interpret the metabolic potential of the permafrost microbiome at an unprecedented scale. Thanks to the long‐read length of the nanopore data set (Figure 1B), compared to hybrid assembly, 42% more of the permafrost microbiome could be annotated at the genus level (Supporting Information: Figure S4A). Also, the KO categories involved in nitrogen and methane metabolism (the main research object in this study) were enlarged by 58% with our strategy (Supporting Information: Figure S4B). Correspondingly, the metabolism network based on the assembly‐free long‐read metagenomic method showed a much higher diversity of nitrogen/methane metabolizing functional populations (Supporting Information: Figure S5).

### Microbial community in thawed permafrost across different altitudes

First, we assessed the repeatability of an experiment by separately sequencing two independent HP4000 samples using MinION (one sequenced on‐site, one sequenced in the lab). The community composition of these two samples was compared at the genus level. A highly similar community structure was observed with Pearson correlation analysis showing an *R* ^2^ value of 0.99 (*p* < 2.2e−16, Supporting Information: Figure S6), suggesting that our sample collection, storage during transportation as well as sequencing handling were reliable. Thus, the comparisons on community shift among vertical alpine and between thawed and frozen should be safe from artificially introduced bias.

**Figure 2:**
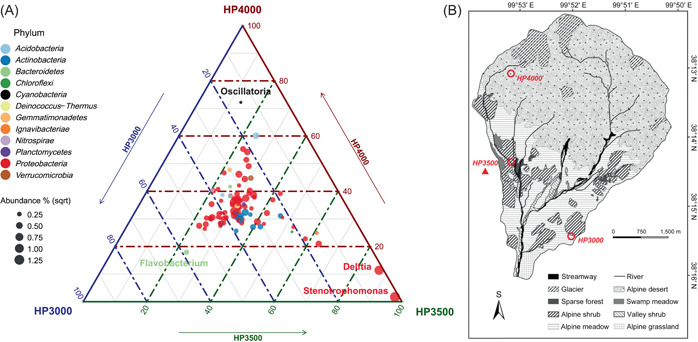
(A) Ternary plot depicts different relative abundances of the genus (>0.5‰) across three altitude samples. Each point represents one genus. The position of each point is determined by the contribution of the indicated compartment to the total relative abundance, and its size represents the average relative abundance across all the three altitude samples. The points are colored according to Phylum affiliation. The significantly enriched genus at different altitude samples was labeled with its taxonomy (namely *Flavobacterium*, *Delftia*, *Stenotrophomonas*, and *Oscillatoria*). (B) The study area and sampling sites in the Qilian Mountain. The figure was modified from the previous study by Chen et al. [69]. The thawed active layer of permafrost soil samples was collected at HP3000, HP3500, and HP4000. The red triangle represents the frozen permafrost samples collected in HP3500.

Metagenomic analysis revealed that the core community of permafrost was conserved along the altitude gradient (Figure 2); 78.4% (719 out of 915) of the genera observed were shared in all permafrost samples at different altitudes (Supporting Information: Figure S7), the abundance of shared genera accounted for more than 99% of the total annotated genera. Next, to further explore the community differentiation, abundant genera (relative abundance > 0.5‰ in any sample, Figure 2A) were compared; 93.8% of these dominant genera were affiliated to *Proteobacteria* and *Actinobacteria*, consistent with previous reports on permafrost community [13,18]. Ternary plot depicting community differentiation (Figure 2A) shows that most dominant genera were clustered in the center of the triangular graph, revealing their similar prevalence in communities at different studied altitudes. In contrast, the physicochemical factors, such as organic carbon, nitrogen, and phosphorus showed evident variation along with altitude ingredients (Supporting Information: Table S1), indicating that the native prokaryotic microbial community in high‐altitude permafrost was recalcitrant to changes in soil chemical content. Such a stable core‐community structure against physicochemical shift was consistent with some previous studies in a high‐altitude environment [32,33].

Despite the conservative nature of the core community, it is interesting to notice that several genera were evidently enriched by regional environmental factors among vertical alpine: *Flavobacterium* enriched in HP3000 sample; *Stenotrophomonas* and *Delftia* enriched in HP3500 sample; and the *Oscillatoria* enriched in HP4000 sample. *Oscillatoria*, enriched in the HP4000 sample, belong to *Cyanobacteria* phylum that obtains their energy through photosynthesis and can survive in the high‐altitude, low‐latitude location where the solar radiation is strong [34]. The functional analysis further confirmed a higher relative abundance of carbon fixation genes in *Cyanobacteria* in the HP4000 sample (Supporting Information: Figure S8). In addition to the Calvin cycle, genes are involved in some additional carbon fixation pathways, such as the reductive citrate cycle (Arnon‐Buchanan cycle), the dicarboxylate‐hydroxybutyrate cycle, and the 3‐hydroxypropionate bi‐cycle, also have been detected with high relative abundance at high‐altitude permafrost. These detected multiple pathways of CO_2_ fixation may imply a variety of light‐utilizing strategies involved in *Cyanobacteria* in the high‐latitude permafrost. In high solar radiation areas, *Cyanobacteria* usually was the major light‐utilizing organisms [35], and support substantial and diverse populations of heterotrophic microorganisms [36].

**Figure 3:**
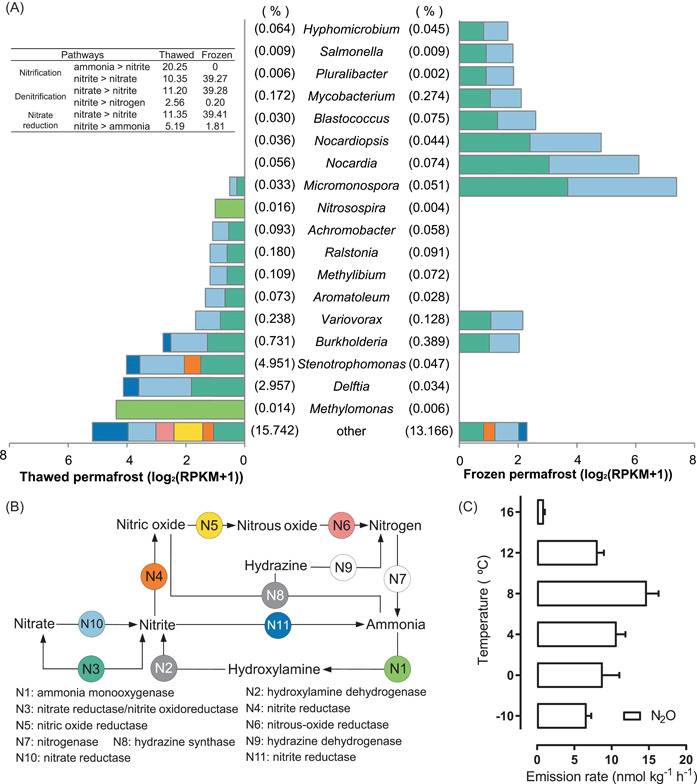
The transcriptional activity of nitrogen metabolism in thawed and frozen permafrost soil at HP3500. (A) The transcriptional activity of different microorganisms involved in nitrogen metabolism. The left and right side of the figure respectively represents activities in thawed and frozen permafrost soil. The values in parentheses represent the relative abundance of microorganisms in the community. Table on the top left corner summarizes the total transcriptional activities of different pathways: ammonia => nitrite: N1 + N2, nitrite => nitrate: N3, nitrate => nitrite: N3 + N10, nitrite => nitrogen: N4 + N5 + N6, nitrite => ammonia: N11. (B) Nitrogen metabolism pathway and key genes. The different colors of key genes are correlated with that in (A). Genes only detected in the unclassified genus are colored in “gray,” while genes undetected are colored in “white.” (C) The N_2_O emission rate at different temperatures in incubation experiments as validation for the field measurement.

Additionally, *Stenotrophomonas* and *Delftia*, respectively taking 4.95% and 2.96% of the community were enriched in the HP3500 sample, where the highest nitrate‐nitrogen concentration of 320 mg kg^−1^ was observed (Supporting Information: Table S1). A previous study showed that *Stenotrophomonas* has an important ecological role in the nitrogen cycles [37]. It can utilize NH_4_^+^, NO_2_^−^, and NO_3_^−^ through assimilation, but it cannot convert them to nitrogen. In addition, it can fix nitrogen when N_2_ is the sole nitrogen source [38]. Our RNA‐seq data of thawed permafrost microbiome also revealed its active involvement in nitrate ammonification (Figure 3), expending metabolic potentials of this interesting soil *genus* and highlighting the influence of nitrogen content on the permafrost community. According to the previous studies, *Delftia* could reduce nitrate to nitrite [39]. The nitrate reduction genes *nar* of *Delftia* are involved in denitrification, dissimilatory nitrate reduction to ammonia (DNRA), and complete nitrification pathways [40] (Supporting Information: Figure S8). Such nitrate reduction ability of *Delftia* was found to be coupled with the degradation of some organic compounds [41]. It is intriguing to notice that neither of these two most dominant nitrate metabolizing genera was able to perform complete denitrification, implying that the nitrate content of permafrost tends to be directly reduced into ammonia other than being released into the atmosphere in form of nitrogen gas, which may, in turn, facilitate the accumulation of nitrogen content in the soil.

### Microbial activity shifts within thawed and frozen permafrost

With the aid of the assembly‐free long‐read metagenomic approach, we were able to interpret the metabolic potential of the permafrost microbiome at an unprecedented scale. The full functional profile of the thawed permafrost at three altitudes is shown in Supporting Information: Figure S9. In particular, we focused on the transcriptional activities of nitrogen and methane metabolism, which were the predominant subcategories in the energy metabolism and played important roles in the biogeochemical cycle, in thawed and frozen permafrost at HP3500.

#### The nitrogen cycles

Figure 3 shows the transcriptional activities (Reads Per Kilobase of transcript per Million mapped reads [RPKM]) of the main nitrogen pathways in HP3500. Overall, the complete nitrification, denitrification, and DNRA pathway were all detected in thawed permafrost soil, while the denitrification pathway in frozen permafrost soil was incomplete. There were no complete pathways of nitrogen fixation but the Anammox pathway could be detected in both thawed and frozen soils. Previous studies showed that the availability of simple labile substrates gradually decreases over time because there is no influx of new energy sources in the permafrost environment [17], and this cold and energy‐limited environment makes the detrital biomass a C and N source for recycling and use [42,43]. Accordingly, no microbial nitrogen fixation could be detected in this study and it is unsurprising to find that the DNRA pathway showed the highest transcriptional activity in both thawed and frozen permafrost soil, especially genes are involved in nitrate to nitrite conversion (N3 and N10 in Figure 3). Unlike denitrification, the DNRA acts to conserve bioavailable nitrogen in the system, producing soluble ammonium rather than unreactive dinitrogen gas [44]. The DNRA process represents a way of preventing the loss of nitrogen from the soil system by microorganisms [45]. The DNRA process can be coupled with the oxidation of electron donors (such as organic matter, hydrogen, sulfide, methane, and iron), and DNRA seems to be more favored than denitrification when the electron donor is in excess relative to nitrate [40]. The observed intense DNRA pathway suggests that the nitrogen metabolism in permafrost tends to be a closed cycle, rather than releasing nitrogen into the atmosphere like some other soil microbiomes, such as fertilized soil [46] and river sediment [47]. The overall transcriptional activity of the DNRA pathway of frozen permafrost (RPKM of 41.22) was higher than that of thawed permafrost soil (RPKM of 16.54), suggesting intense microbial activity in the frozen state. Previous studies also revealed that there are still active microorganisms in cold habitats, such as glaciers [48] and permafrost [49].

Even though the overall nitrogen cycle is closed in high‐altitude permafrost, the increased activities of microbial denitrification were observed in the thawed permafrost topsoil as evidenced by the upregulated transcriptional activities of key genes (Figure 3). Since denitrification is a typical heterotrophic process, the increased availability of carbon in thawed permafrost could serve to facilitate its prevalence in permafrost. The gas‐phase measurement of the thawed permafrost in the field (about 15°C) showed a slow consumption rate (18.8 nmol m^−2^ h^−1^, Supporting Information: Figure S10) of N_2_O, a powerful greenhouse gas, whose generation could be resulted from the increased denitrification activities [50]. Meanwhile, the obvious transcriptional activity of N_2_O reduction was observed in thawed permafrost, but not in frozen permafrost (Figure 3). These results suggested N_2_O was more likely to be consumed by microorganisms and prone to convert to N_2_ in thawed permafrost with a temperature of 15°C. This phenomenon can be further proved by our soil incubation experiment that the N_2_O emission increased with the thawing of permafrost soil from −10°C to 8°C, and N_2_O emission decreased as the temperature continued to rise to above 10°C (8°C to 16°C) (Figure 3C). This is mainly because the low‐temperature environment inhibits N_2_O consuming enzymes (N_2_O reductase) to a greater extent than the N_2_O producing enzymes (NO_3_^−^, NO_2_^−^, and NO reductase) [51], and the revived activity of N_2_O reductase above 10°C caused decreased N_2_O emission observed in an incubation experiment.

Additionally, the microbial community involved in nitrogen metabolism showed an evident shift between thawed and frozen states. Studies on Arctic/Antarctic permafrost showed the effect of being frozen is the critical determining factor on microbial community and function [17]. The thaw of active layer permafrost will cause rapid shifts in the microbial and functional genes, including the C and N metabolism genes [19]. During thawing, the activity of oligotrophic microorganisms decreases and the copiotrophic activity increases [18], suggesting that the frozen trapped organic matter becomes accessible and provide an energy source for microorganism thawing [9,19]. These results helped explain our observation that the transcription of denitrification metabolism in thawed permafrost soil was more active (Figure 3) since most denitrifiers are heterotrophic. Accordingly, microbes involved in the DNRA pathway shifted from copiotrophic/heterotrophic members (such as *Delftia*, *Stenotrophomonas*, and *Burkholderia*) of *Proteobacteria* in thawed permafrost soil, to oligotrophic/autotrophic members (such as *Micromonospora*, *Nocardia*, and *Nocardiopsis*) from *Actinobacteria* in frozen permafrost soil [18].

*Nitrosospira* were the active ammonia‐oxidizing bacteria (AOB) within the community of thawed permafrost, while no AOB could be identified in frozen permafrost soil, suggesting clear repression of AOB activity in frozen permafrost. We could not detect active ammonia‐oxidizing archaea (AOA) in permafrost and such AOB‐dominated nitrogen metabolism was also observed in research in cold environments like high‐altitude lakes [52] and permafrost in northeast Siberia [53]. *Nitrosospira* is a typical AOB and lives in various environments [54,55]. Although typical *Nitrosospira* like those in domestic wastewater treatment plants prefers warmer growing environment (25°C to 30°C) [56], a recent study had found certain species of *Nitrosospira* could adapt and enrich in the cold environment [53], and our results consolidated this finding. Additionally, *Methylomonas* is the plausible AOB in permafrost. *Methylomonas* is an aerobic methanotroph, which obtains its carbon and energy from methane [57]. During thawing, the organic matter in the active layer permafrost was decomposed to form methane [12], which may facilitate the enrichment of species of *Methylomonas*. Previous studies showed that *Methylomonas* encodes a sequence‐divergent particulate methane monooxygenase and it also could possibly be involved in ammonia oxidation [58,59].

#### Active methane oxidation in thawed permafrost

**Figure 4:**
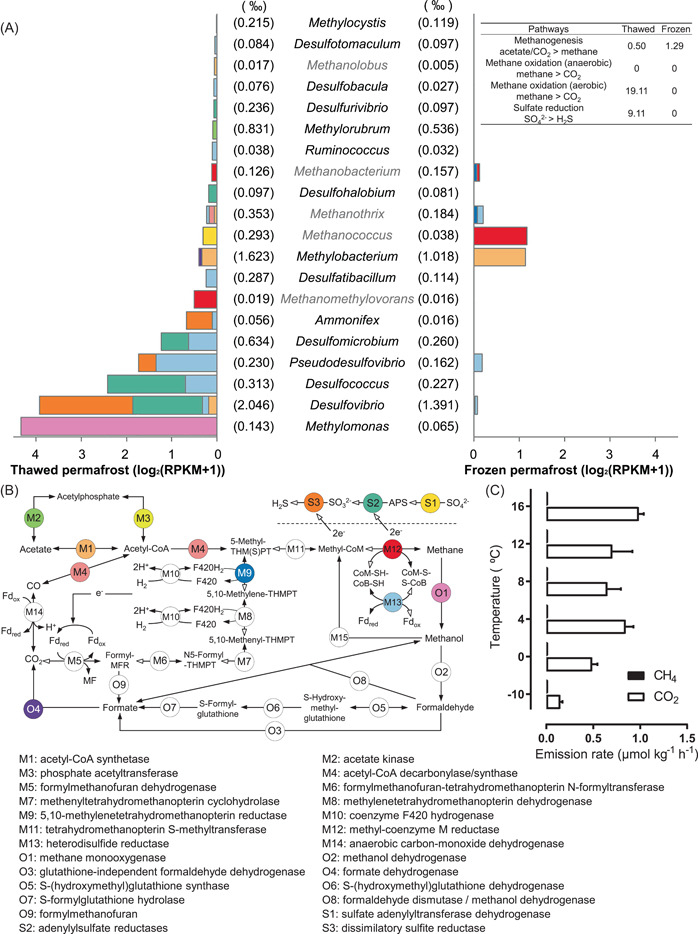
The transcriptional activity of methane metabolism in thawed and frozen permafrost soil at HP3500. (A) The transcriptional activity of different microorganisms involved in methane metabolism. The left and right side of the figure respectively represents activities in thawed and frozen permafrost soil. The values in parentheses represent the relative abundance of microorganisms in the community. The table on the top right corner summarizes the total transcription activities of major methane metabolizing pathways: methanogenesis: the sum of M12 in methanogens, methane oxidation (anaerobic): the sum of M12 in anaerobic archaeal methanotrophs (order *Methanosarcinales* [ANME‐2 and ANME‐3] and “Ca. *Methanophagales*” (ANME‐1), methane oxidation (aerobic): the sum of O1–O9, sulfate reduction: the sum of S1–S3. (B) Pathways and key genes of methane metabolism. The different colors of key genes are correlated with that in (A). Genes undetected are colored in “white.” The hollow arrows indicate the direction of anaerobic methane oxidation. (C) The CO_2_ and CH_4_ emission rates at different temperatures in incubation experiments as validation for the field measurement.

Since some enzymes in methane metabolism catalyze reactions across multiple pathways [17], we identified these specialized methane metabolic microorganisms with manual filtering according to previous studies [60–63]. As shown in Figure 4, no complete pathways of methanogenesis and methanotrophy could be detected, which was probably caused by the low abundance of methanogens and methanotrophs in permafrost (about 0.8‰ in thawed permafrost and 0.4‰ in frozen permafrost). Nevertheless, methyl‐coenzyme M reductase (Mcr), a key gene catalyzing the rate‐limiting and terminal step of methanogenesis and the reverse reaction in archaeal methanotrophs [63], was vigorously transcribed in several methanogenic genera, suggesting the active methanogenic activity in permafrost.

The methanogenic community and pathways showed evident variation between thawed and frozen permafrost. The main active methanogens shifted from *Methanomethylovorans* (order *Methanosarcinales*, which perform both aceticlastic and hydrogenotrophic methanogenesis [63,64]) in thawed permafrost soil to *Methanococcus* (order *Methanococcales*, strict hydrogenotrophic methanogens [63,65]) in frozen permafrost soil (Figure 4). Further, the methanogenic pathways in thawed permafrost were more acetate‐dependent with active enzymes detected in *Methanothrix* (M1, M4, and M13) and *Methanolobus* (M1), and both of them were affiliated to order *Methanosarcinales* (Figure 4B). However, strict hydrogenotrophic methanogens, *Methanococcus* and *Methanobacterium* [65], were more prevalent in frozen permafrost, and the methanogenesis pathway of *Methanosarcinales* also switched from aceticlastic to H_2_‐dependent (M9, M13 of *Methanothrix*, Figure 4A right‐side) in the frozen state. Our metatranscriptional results further confirmed the previous speculation that permafrost thawing will facilitate a shift from hydrogenotrophic to aceticlastic methanogenesis [66]. The higher concentration of accessible organic matter for microbial activity [17], and the active acetogenesis facilitated by polysaccharide degradation in thawed active layer permafrost [12], could help to explain the observed shift from hydrogenotrophic to aceticlastic methanogenesis in thawed Qilian Mountain permafrost.

Aerobic methane oxidation (AMO) was the major methanotrophy process in Qilian Mountain and the active AMO was only detected in thawed permafrost soil (Figure 4). The active AMO was mainly attributed to *Methylomonas* (Figure 4). Recent findings had shown that microaerobic environments can provide sufficient oxygen for the growth of *Methylomonas* [67]. And *Methylomonas* encodes a sequence‐divergent particulate methane monooxygenase, which could possibly catalyze ammonia oxidation [58,59]. The active involvement of *Methylomonas* in both ammonia and methane oxidation observed in this study indicated a plausible synergy between the carbon and nitrogen cycle in the permafrost microbiome. In addition to AMO, anaerobic methane oxidation (AnMO) can also oxidize methane to CO_2_ by reversing the hydrogenotrophic methanogenesis pathway. These anaerobic methanotrophs usually belong to the order of *Methanosarcinales* (ANME‐2 and ANME‐3) and “*Ca. Methanophagales*” (ANME‐1) [63], and AnMO reactions are needed to couple with syntrophic bacterial partners who could provide electrons by reducing sulfate, nitrate, or irons [62]. However, in this study, anaerobic archaeal methanotrophs were not detected although high activities of nitrate (Figure 3) and sulfate (Figure 4) reduction were observed. Such absence of AnOM was consistent with a previous study on the Arctic thawing permafrost [12].

Overall, the low methane production to consumption ratio of 1:38 (estimated based on transcriptional activity of key genes) was observed in the thawed permafrost, suggesting that microbial AMO can effectively transform CH_4_ to CO_2_ and mitigate CH_4_ release from thawed permafrost topsoil. This speculation was consistent with the low CH_4_ emission rate of 2.0 μmol m^−2^ h^−1^ observed in the gas phase on‐site, while the CO_2_ emission rate was 50 times higher (104.5 μmol m^−2^ h^−1^, Supporting Information: Figure S10). Additionally, the incubated permafrost under different temperatures also showed that more CO_2_ was released with the thawing of permafrost (Figure 4C). Since CH_4_ was a much stronger greenhouse gas than CO_2_ [68], the active bioconversion of CH_4_ by *Methylomonas* could relieve greenhouse gas emissions initiated by permafrost thawing.

## CONCLUSIONS

To address the large data loss caused by the metagenomic assembly, here we introduce FUNpore, a long‐read correction and annotation framework. Such a correction‐based annotation strategy showed promising precision and recall in functional annotation and could provide more permafrost microbial information. On‐site MinION metagenomic sequencing of high‐altitude permafrost soil showed that although 78.4% of genera were shared among vertical alpine, nitrate concentration and solar radiation can lead to a certain level of community differentiation. The active nitrogen metabolism in permafrost tends to be a closed cycle, especially in frozen permafrost. Increased denitrification activities were also observed in thawed permafrost, but N_2_O generated during the thawing process was more likely to be converted to N_2_. The active methanogenesis switched from H_2_‐dependent in frozen permafrost to acetate‐dependent in the thawed state, and the vigorous aerobic methane oxidation by *Methylomonas* could greatly mitigate CH_4_ emissions in thawed permafrost. Collectively, in the context of global warming, the increasing thawed state will evidently change the nitrogen and methane metabolism in permafrost, and microorganisms could serve as bio‐filters to relieve greenhouse gas emissions caused by permafrost thawing.

### Limitations and cautions

In this study, we integrated long‐read‐based metagenomics and RNA‐seq to reveal microbial responses to the thaw‐frozen cycle in high‐altitude permafrost. Even our correction‐based annotation strategy seems to work well in microbial activities identification and our results correlated to other permafrost microbiome work [12,18], some limitations may affect our results. First, the current pattern between the thawed‐frozen cycle was based on soil samples collected in 1 year. A long‐term study would be needed to consolidate the pattern. Second, even functional annotation using an alignment‐based method (BLASTP of Prokka) was mostly accurate, Hidden Markov Model (HMM)‐based tools showed many false annotations. Therefore, popular tools based on HMM, such as GroupM, HMMer, Pfamscan and so on, shall not be used to predict the function of postcorrected nanopore reads. Additionally, since nanopore reads were virtually much longer than assembled contigs, the correction‐based strategy could resolve functionalities of microbes down to the genus level at which primary metabolic capacities are usually conserved. However, further genome binning of the postcorrection nanopore reads will be needed to study microbial functionality that tends not to conserve at the genus level, such as pathogenicity and antibiotic resistance.

## METHODS

### Study area and permafrost sample collection

The sampling campaigns were carried out in the Qilian Mountain (N38°13′−38°16′, E99°50′−99°53′), which is located in the northeastern of the Tibet‐Qinghai Plateau, and the altitude of this mountain is about 4800 m above sea level [69]. The mean annual temperature is about −5–11°C, and the highest average daily temperature is about 23°C in July and the lowest average daily temperature is about −20°C in January. The perennial low temperature makes the accumulated snow on the mountain form a wide range of glaciers. The active layers of permafrost at different altitudes (3000, 3500, and 4000 m) were chosen as sampling sites, namely HP3000, HP3500, and HP4000. The sampling campaigns were carried out in August 2018 to collect thawed permafrost soil at three sampling sites, and the frozen permafrost soil was collected at HP3500 in November 2018. The permafrost soil samples were collected from the surface of soil pits formed by the seasonal freeze‐thaw process. Each soil sample was collected from three or more nearby locations and sealed in 50‐ml sterilized polypropylene tubes after being well mixed. The soil samples from each site were divided into three parts: one part was immediately used to extract DNA for Oxford Nanopore sequencing in the field; the second part (around 5 g) was immediately stabilized by soaking in 50 ml RNA later (Invitrogen) for RNA extraction back in the lab and the last part was stored in ice and transported to the laboratory for other subsequent analysis. The methods for physicochemical properties analyses on permafrost soil samples were described previously [70]. The physicochemical properties are listed in Supporting Information: Table S1.

### DNA extraction and MinION sequencing in the field

The soil samples were used to extract DNA from three biological duplications using the DNeasy PowerSoil Kit (Qiagen) according to the manufacturer's protocols. DNA concentrations were determined by Qubit 2.0 instrument (Thermo Fisher Scientific). Equal amounts of DNA from each duplication were pooled and purified using AMPure XP beads (Beckman Coulter).

For each sample, about 2.0 μg purified DNA was used to perform sequencing library preparation using SQK‐LSK108 1D ligation genomic DNA kit following the manufacturer's protocol with minor modifications. Briefly, approximately 1.5 μg metagenomics DNA was in 200 μl polymerase chain reaction (PCR) tubes. We used miniPCR (DNA Discovery System™) to incubate those tubes at 30°C for 5 min and then to 80°C for another 3 min. We doubled the incubation time for adaptor ligation on purpose for better performance based on our previous experience. MinION sequencing was performed using R9.4 flow cells (FLO‐MIN 106) on a laptop computer with a dual‐core CPU, 16 G RAM, and 1 T SSD. To ensure genome coverage of the permafrost community, only one sample was loaded for each flow cell in the field sequencing. Fast5 files generated by MinKNOW were transformed to fastq files using Guppy basecaller v 3.0.3 (https://github.com/nanoporetech/pyguppyclient), and the adapters on the ends and middle of reads were trimmed using Porechop v0.2.4 (https://github.com/rrwick/Porechop). A total of five flow cells were used to run independent MinION sequencing for all five samples, including one biological duplication. On average, 5.5 Gb clean Nanopore reads (fasta format) were obtained for each sample (Supporting Information: Table S2).

### Gas sample collection in the field

To determine the fluxes of N_2_O, CO_2,_ and CH_4_ at the soil‐air interface, the gas exchange rates were measured by the static chamber method modified from the previous study [71]. Briefly, two chambers equipped with vent holes were placed on the soil to collect gas simultaneously at a 20 min interval for 80 min. In the beginning, the chamber was carefully placed on the soil and allowed to equilibrate the atmospheric pressure for about 60 s. Then, 25 ml of gas was extracted from the chamber using a 100 ml polypropylene syringe equipped with a two‐way valve and transferred to pre‐evacuated exetainers (12 ml) after 5 ml sampled gas was discharged to rinse the valve and syringe needle. Finally, the parallel gas samples collected from chambers at 0, 20, 40, 60, and 80 min were transported to the laboratory for the detection of N_2_O, CO_2,_ and CH_4_.

### RNA extraction in the laboratory

The cosampled permafrost samples were immediately stabilized in RNA later and transported to the laboratory on ice. Then, the total RNA was extracted using the RNeasy PowerSoil Total RNA Kit (Qiagen) according to the manufacturer's protocols. The method of measuring and purifying total RNA followed our previous study [72].

### Illumina sequencing

The extracted DNA was used to shotgun metagenomic sequencing after returning to the laboratory. Briefly, about 1 μg DNA was performed to construct a library with a 300 bp insert size, followed by sequencing on Illumina HiSeq. 4000 platform (Magigene Company) with the PE150 strategies. The raw sequences were assigned to each sample according to the unique barcode, and the adapter was cut using Seqprep (https://github.com/jstjohn/SeqPrep). The reads with average quality scores <20 or length <60 bp were removed using Sickle (https://github.com/najoshi/sickle). On average, 44.3 Gb clean reads (fasta format) were obtained for each sample.

The extracted RNA was used to construct the library using NEBNext Ultra II Directional RNA Library Prep Kit for Illumina (New England Biolabs) and sequenced on Illumina HiSeq. 4000 platform (Magigene Company) with the PE150 strategies. After quality control, the ribosomal RNA sequences were removed by riboPicker [73]. On average, 38.5 Gb messenger RNA (mRNA) reads (fasta format) were obtained for each sample. All the raw sequencing data were deposited into the EBI European Nucleotide Archive database under accession number PRJEB38431.

### Nanopore reads correction

We first replaced the bases with extremely low‐quality (quality score lower than 5) with “N.” Then, Pilon (v1.23 https://github.com/broadinstitute/pilon) was used to polish the Nanopore reads by aligning Illumina reads to Nanopore reads using bwa (v0.7.17) [74] alignment.

Since the polished Nanopore reads still suffer a high ratio of frame‐shift error [31], we developed a pipeline, named as FUNpore, to correct the frame‐shift error of nanopore reads. Briefly, a frame‐shift aware DNA‐to‐protein alignment of Nanopore reads is performed against the NCBI Refseq_protein database using LAST [75]. Then, a self‐made script is used to correct the insertion or deletion error in the Nanopore reads based on the location of frameshifts reported in the LAST alignments. As shown in the mechanism of LAST [75], in a frame‐shift alignment, the “\” indicates a forward shift by one nucleotide, and the “/” indicates a reverse shift by one nucleotide. To correct these frame‐shift errors, we insert another previous nucleotide base into the read in the former case and delete the current nucleotide base from the read in the latter case (frame‐shift correction principles illustrated in Supporting Information: Figure S1).

Next, we used two data sets to evaluate the accuracy and recall of functional prediction based on KEGG Orthology (KO) using these postcorrection nanopore reads. The first data set was the nanopore whole genome sequence of 20 isolated strains. The hybrid assembly of the strains was used as a golden standard for evaluation (Supporting Information: Table S3). The second data sets were 315 whole genomes downloaded from the NCBI genome database (Supporting Information: Table S4). To ensure the representativeness of the evaluation, the same number of genomes was chosen for each Phylum. We randomly introduced a 5% error into these downloaded genomes to make a mock nanopore data set. Then the functional predictions of the corrected mock nanopore reads were compared to that of the downloaded genome for evaluation.

### Sequencing data analysis

The taxonomic identification of the frame‐shift corrected reads was based on the combined results of kmer‐based KRAKEN v0.10.6 [76] and alignment‐based Taxator [77]. For Taxalor analysis, consensus phylogeny was retrieved from Megablast [78] alignment against NCBI *nt* database (downloaded on August 8, 2019) using the MEGAN‐like LCA algorithm. Prokka [79] was used to assign KEGG functional categories. The abovementioned taxonomic and functional annotation steps were all packed in our FUNpore framework (https://github.com/sustc-xylab/FUNpore.git). The analysis workflow and performance benchmark are shown in Figure 1A.

The mRNA reads were mapped to ORFs using bwa with “mem” parameter. Based on the bwa alignment, RSEM [80] was used to compute the RPKM. RNA‐RPKM of a given gene represents the overall transcriptional activity of the gene in the community.

### The batch incubation experiments

An amount of 200 g of permafrost soil was placed in a serum bottle with a rubber stopper and continuously incubated across a temperature gradient of −10°C, 0°C, 4°C, 8°C, 12°C, and 16°C with 24 h incubation time for each temperature. The gas samples were collected at the beginning and end of the incubation experiment at each temperature. To balance the vacuum caused by gas sampling, the bottle was flushed with N_2_ after each sampling. Two parallel incubation experiments were conducted simultaneously.

### Gas detection and analysis

The detection of N_2_O, CO_2_, and CH_4_ was determined using a gas chromatograph equipped with a thermal conductivity detector (TCD) following our previous study [81]. The column was HayeSep‐Q and the carrier gas was high pure helium (99.999%) with a flowing rate of 30 ml min^−1^. The operating temperature of the oven and TCD were 120°C and 200°C, respectively.

The emission or consumption rate of specific gas was calculated according to the following formula [71]:

$$R_{\text{gas}}=\frac{∆C}{∆t}\times \frac{\mathrm{PV}}{R\mathrm{TA}},$$

where ∆C/∆t means the slope (ppm/h) of the specific gas accumulation in the chamber. A positive slope value indicates that the soil releases gas, and a negative slope value indicates that the soil consumes gas; *P* means the atmospheric pressure (atm); *V* means the chamber volume (L); *R* means the gas constant (0.0821 L atm K^−1^ mol^−1^); *T* means the gas temperature (K); and *A* means the area of the chamber on the surface of the soil (m^2^) or the weight of incubated permafrost soil (kg).

## AUTHOR CONTRIBUTIONS

Yu Xia and Chenyuan Dang designed this study, and Chenyuan Dang analyzed the data and wrote the manuscript. Yu Xia, Chenyuan Dang, Ziqi Wu, and Yuqin Sun collected the samples. Chenyuan Dang, Ziqi Wu, and Miao Zhang extracted the DNA and RNA. Chenyuan Dang and Ziqi Wu performed metagenomic sequencing using Nanopore technology. Yu Xia developed the FUNpore pipeline, and Chenyuan Dang participated in debugging and improvement. Xiang Li, Yan Zheng, Ren'an Wu, and Yu Xia edited the manuscript. All authors read and approved the final manuscript.

## CONFLICTS OF INTEREST

The authors declare no conflicts of interest.

## Supporting information

Supporting information.

Supporting information.

## Data Availability

All the raw sequencing data were deposited into the EBI European Nucleotide archive (ENA) database under accession number PRJEB38431. The scripts for FUNpore are freely available at https://github.com/sustc-xylab/FUNpore.git. Supplementary materials (figures, tables, scripts, graphical abstract, slides, videos, Chinese translated version, and update materials) may be found in the online DOI or iMeta Science http://www.imeta.science/.
