# Supplementary material for: Microorganisms as bio‐filters to mitigate greenhouse gas emissions from high‐altitude permafrost revealed by nanopore‐based metagenomics
Source: Imeta. 2022 May 8;1(2):e24. doi: 10.1002/imt2.24 (PMC10989947; doi:10.1002/imt2.24)
Supplement: Supplementary file 1 — Supporting information. [file IMT2-1-e24-s002.docx]

**Supplementary materials for**

**Microorganisms as bio-filters to mitigate greenhouse gas emissions from high-altitude permafrost revealed by Nanopore-based metagenomics**

Chenyuan Dang ^a,b^, Ziqi Wu ^a^, Miao Zhang ^a^, Xiang Li ^a,d^, Yuqin Sun ^c,d^, Ren’an Wu ^b^, Yan Zheng ^c,d^, Yu Xia ^a,d^ *

*^a^* School of Environmental Science and Engineering, College of Engineering, Southern University of Science and Technology, Shenzhen, 518055, China

*^b^* Laboratory of High-Resolution Mass Spectrometry Technologies, Dalian Institute of Chemical Physics, Chinese Academy of Sciences (CAS), Dalian, 116023, China

*^c^* Shenzhen Key Laboratory of Marine Archaea Geo-Omics, Department of Ocean Science and Engineering, Southern University of Science and Technology, Shenzhen 518055, China

*^d^* State Environmental Protection Key Laboratory of Integrated Surface Water-Groundwater Pollution Control, School of Environmental Science and Engineering, Southern University of Science and Technology, Shenzhen 518055, China

**Correspondence:**

Yu Xia, PhD, School of Environmental Science and Engineering, College of Engineering, Southern University of Science and Technology, Shenzhen, 518055, China. Email: xiay@sustech.edu.cn


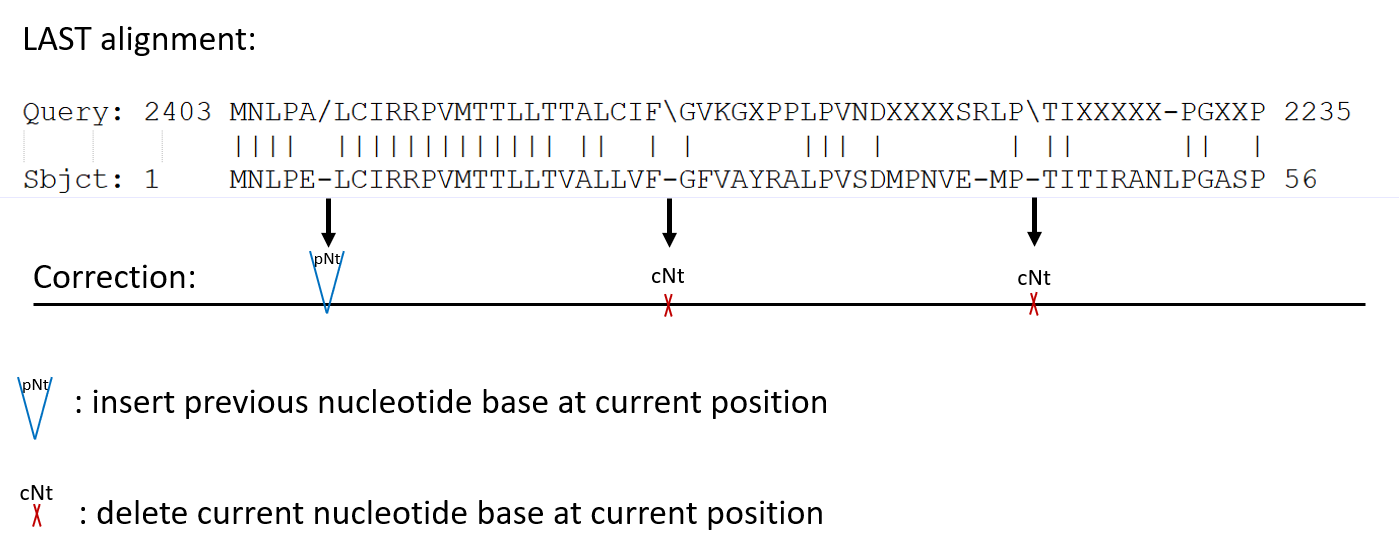
Figure S1. Principle of FUNpore frame-shift correction.

Figure S2. The overview of base quality of Nanopre read and the distribution of Illumina reads mapping on nanopore reads. We used 11 Gb Illumina reads (fasta format) to map nanopore reads (fastq, 2.6 Gb).


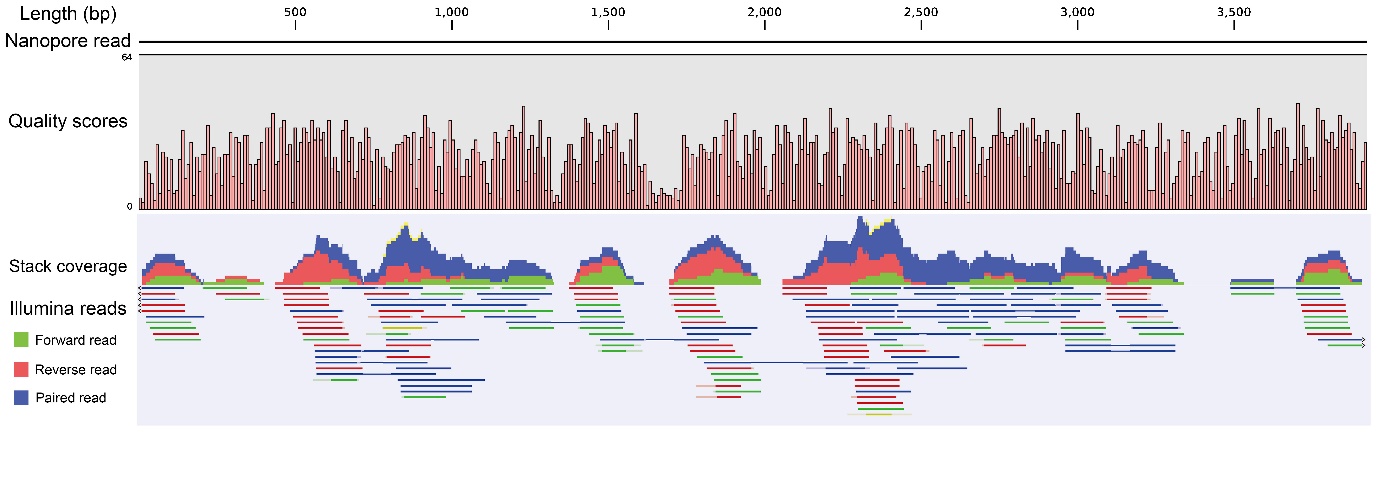


Figure S3. Average read accuracy (**A**) and estimated erroneous base number (**B**) before and after Pilon polish. We randomly selected 15000 nanopore reads and used 10 Gbp (fasta format) Illumina reads to polish nanopore reads using Pilon.


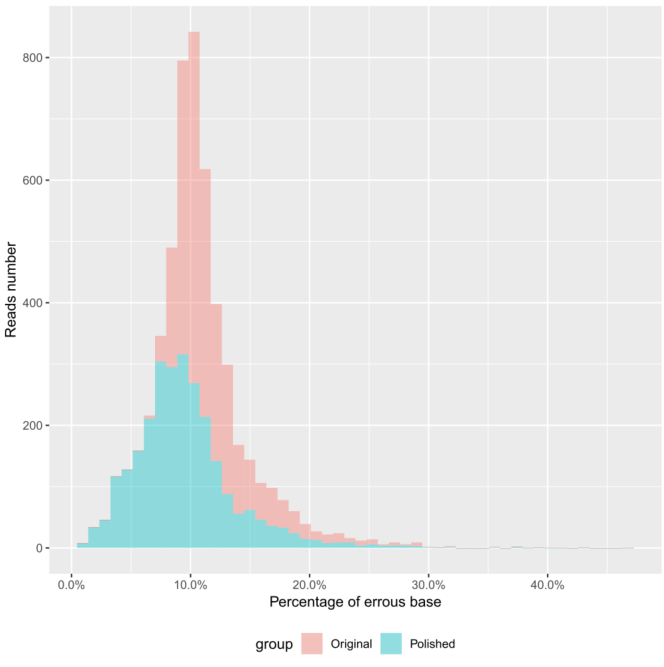


**B**


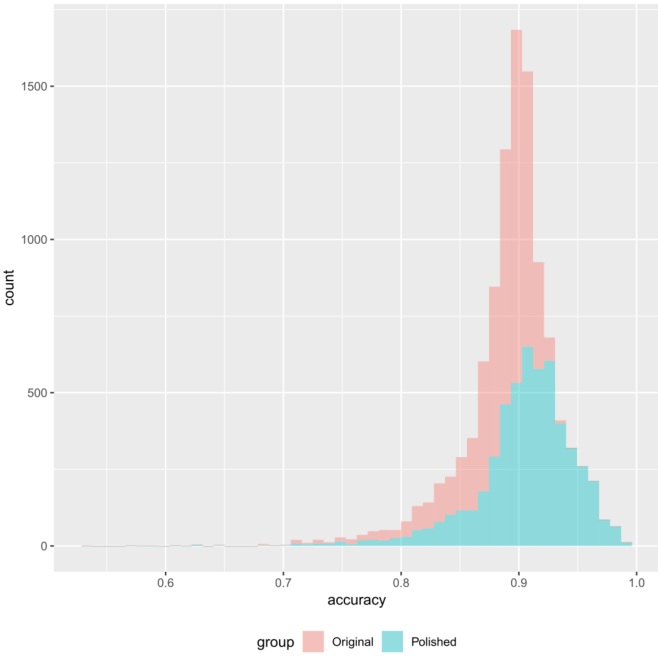


**A**


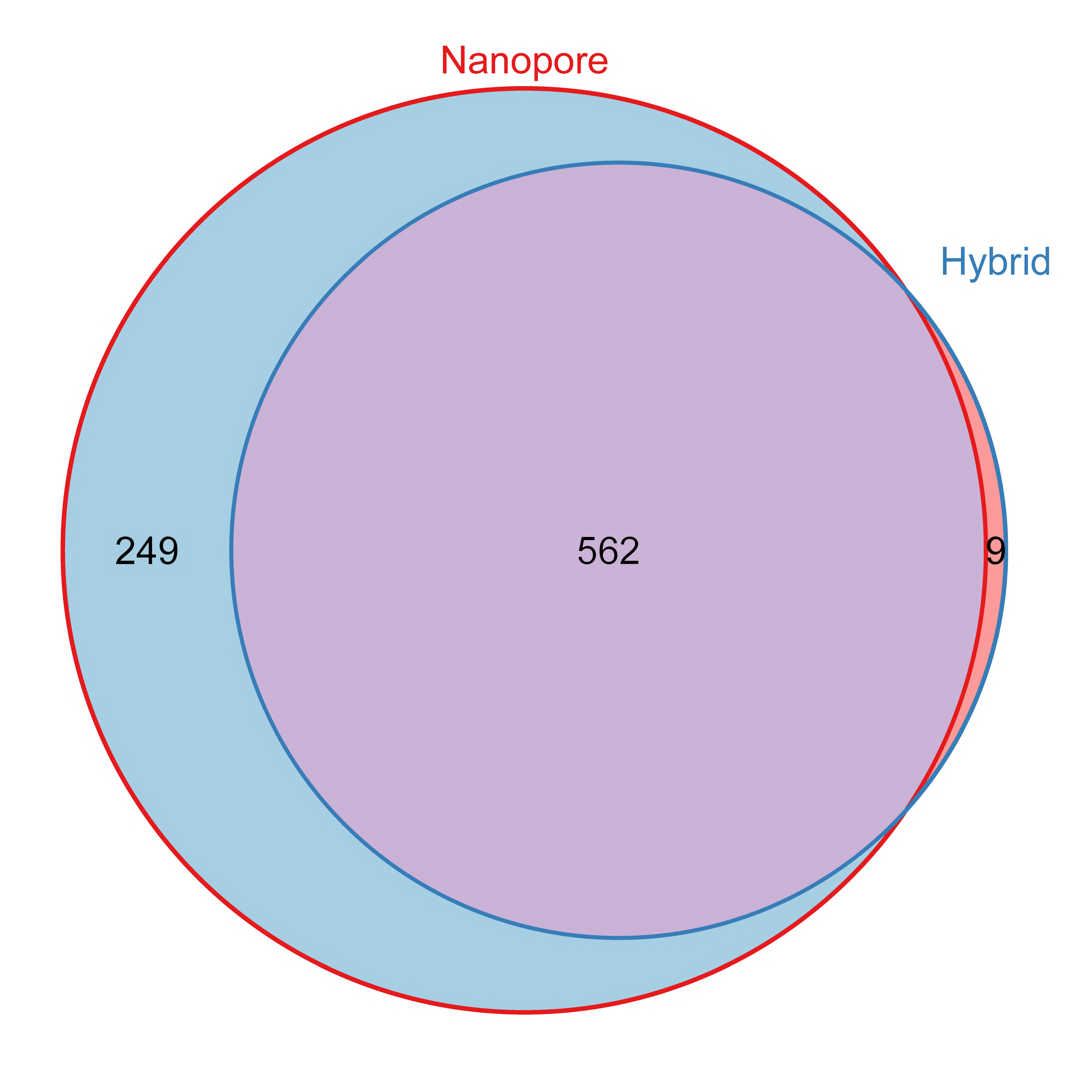


**A**


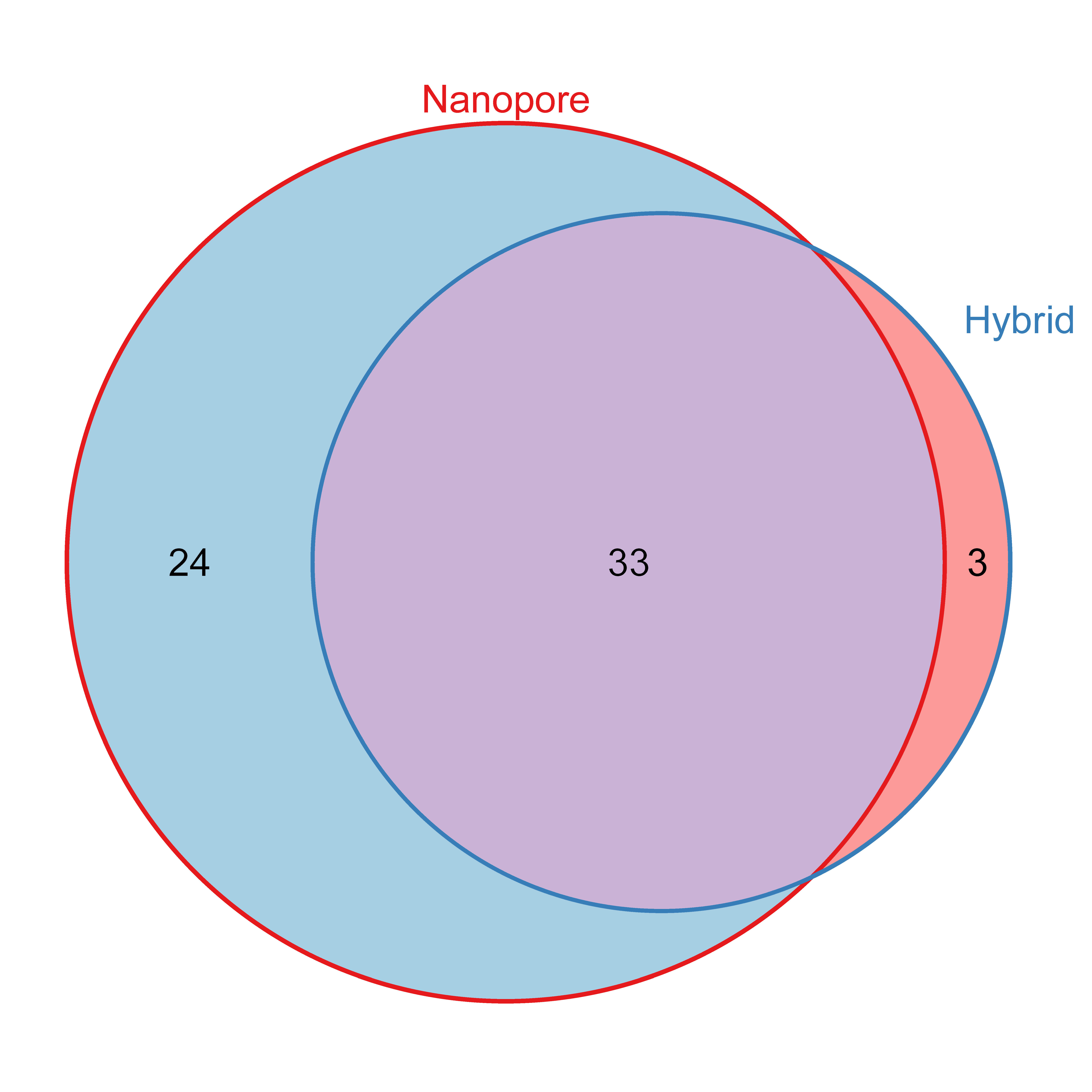


**B**

Figure S4. The shared genus (**A**) and KO involved in nitrogen and methane metabolism (**B**) number between assembly-free long-read metagenomic and hybrid-assembly approach.


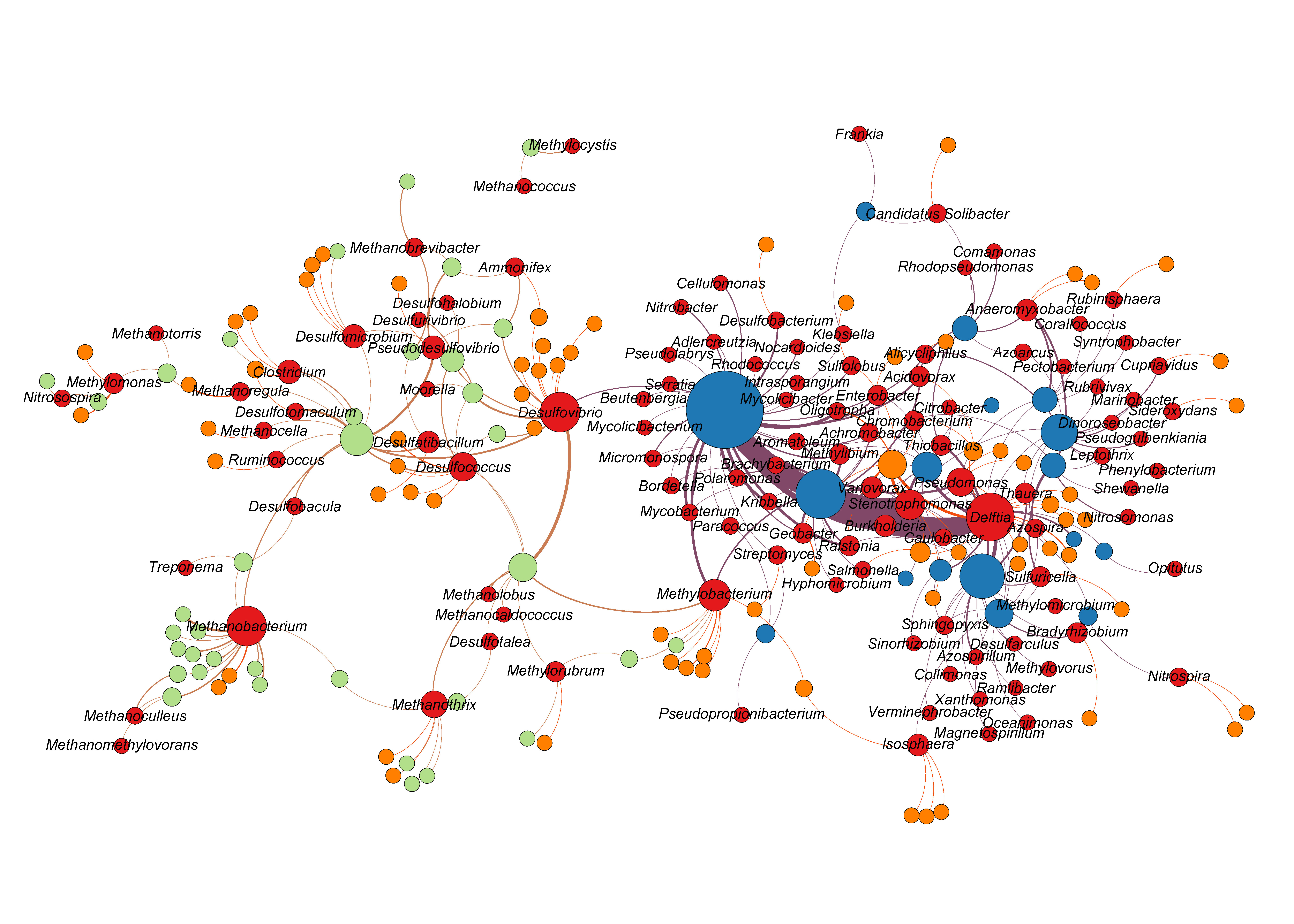


**A**


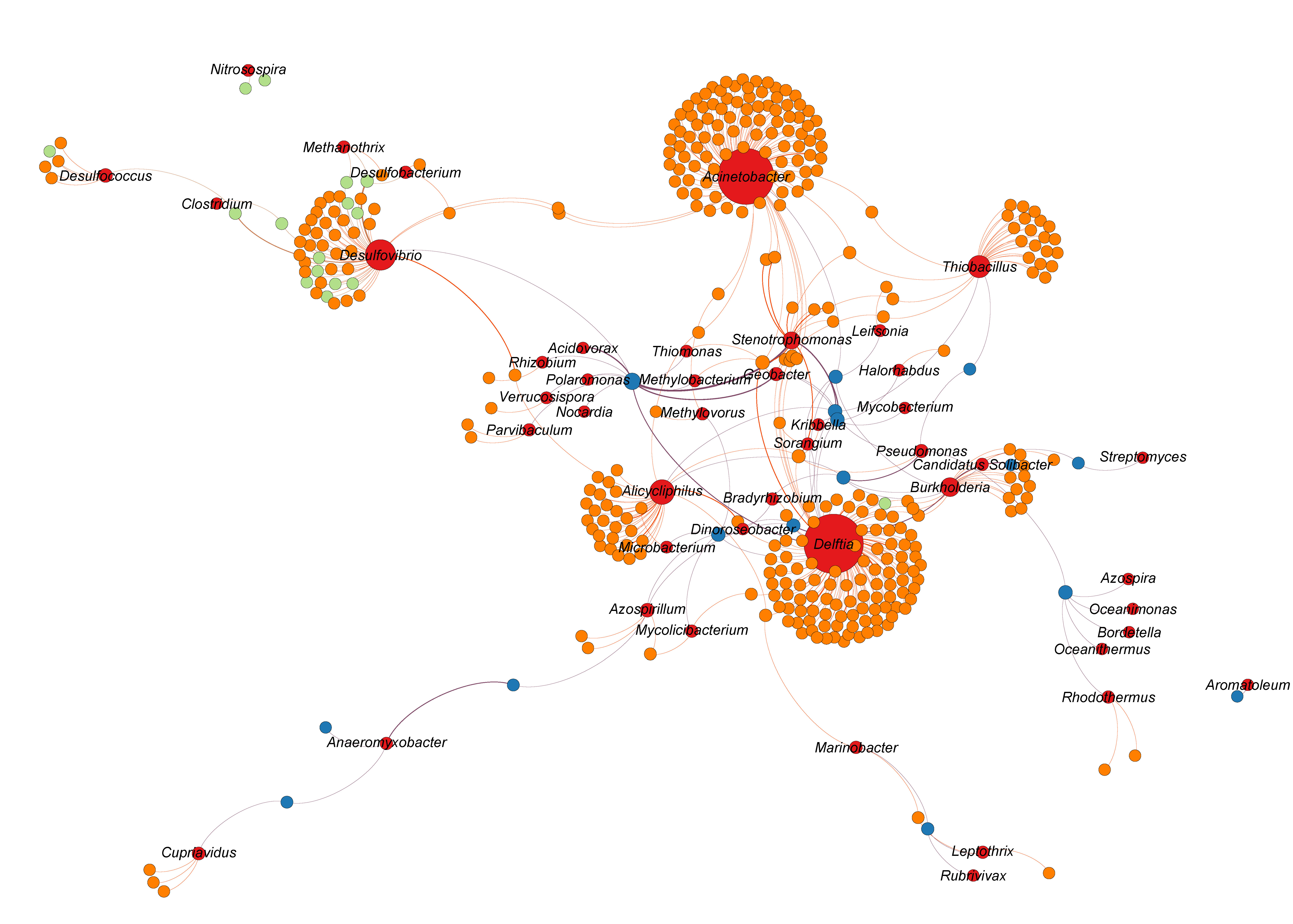


**B**

Figure S5. The network analysis of genus and KO categories annotated by assembly-free long-read metagenomic (**A**) and hybrid-assembly approach (**B**). red nodes: genera involved in nitrogen and methane metabolism. Blue and green nodes respectively represent genes involved in nitrogen and methane metabolism, while orange nodes represent other genes identified in the community.


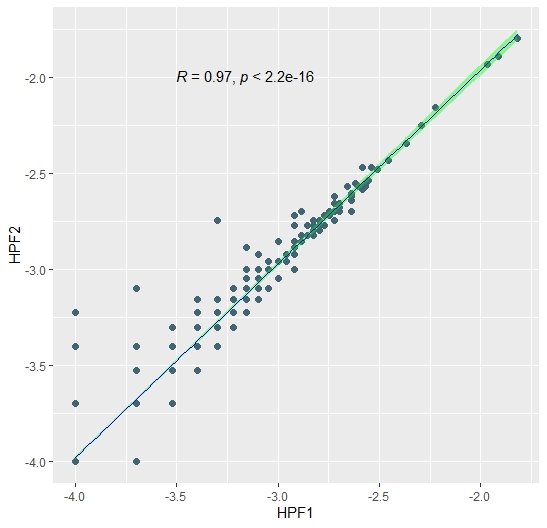
Figure S6. Biological repetition results at genus level. The relative abundance of genera was transformed by log10. The two biological permafrost samples were collected at HP4000 and were sequenced respectively using two MinION R9.4 flow cells (FLO-MIN 106).


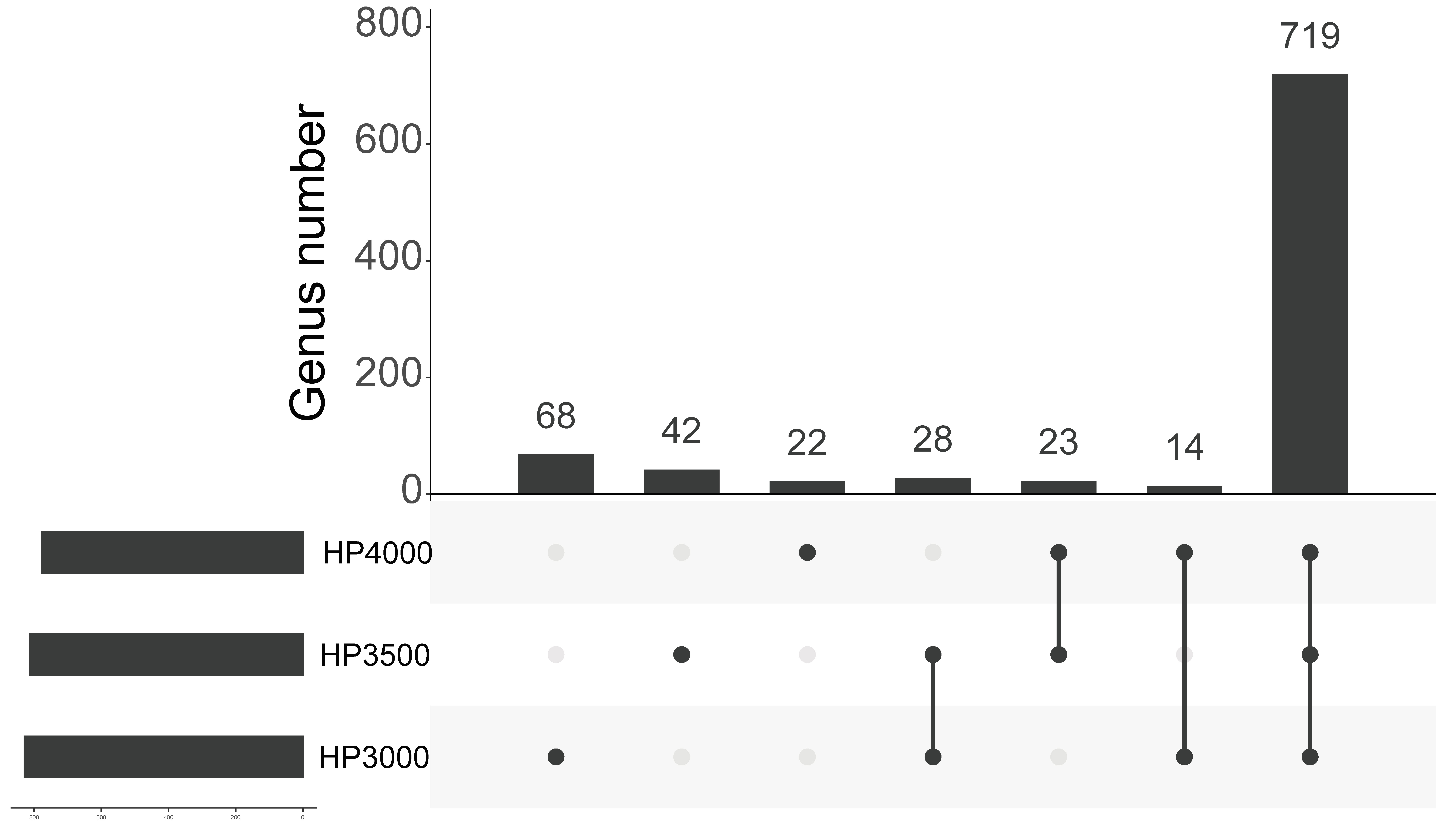


Figure S7. The shared genus number among thawed permafrost soil samples at different altitudes.


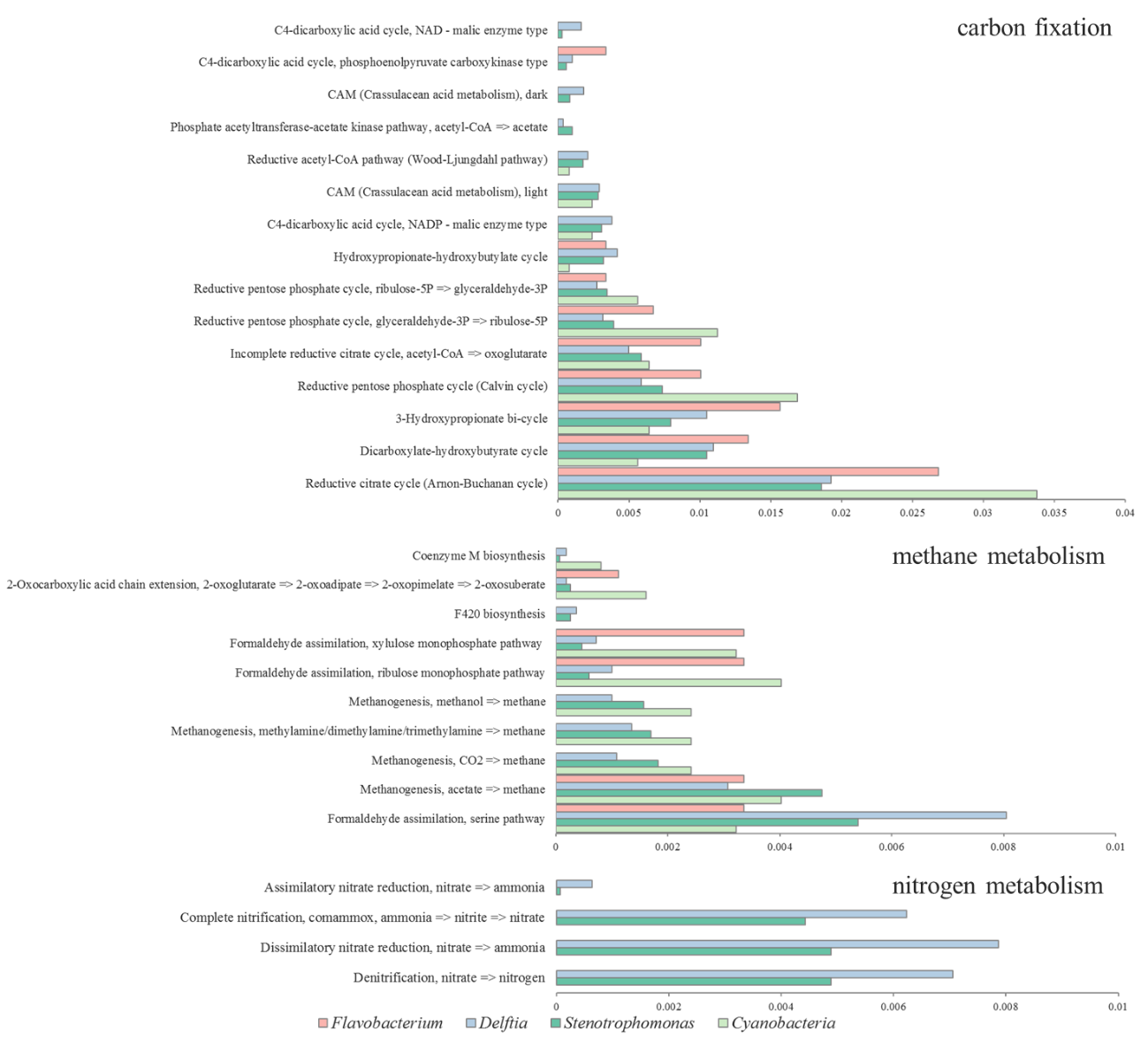
Figure S8. Metabolic potentials of enriched genera at different altitude samples based on the relative abundance of key genes involved in carbon and nitrogen metabolism. Genes of *Flavobacterium*, *Delftia* and *Stenotrophomonas* are extracted at genus level, and the genes of *Oscillatoria* are extracted at phylum level because of its low abundance.

Figure S9. **A.** The full profile of the permafrost function. KEGG annotation analysis showed that the genes involved in metabolism were the most abundant, accounting for 48.8%, 47.3% and 47.9% of total genes in HP3000, HP3500 and HP4000 sample, respectively. **B.** The second abundant category was the genetic information processing, which accounted for 15.8%, 16.4% and 16.6% across the different altitude samples (Figure S2). Some subcategories, such as carbohydrate, amino acid, energy and nucleotide metabolism as well as translation, were detected with high relative abundance. This was similar to previous studies that these housekeeping genes related to maintenance of basic cellular functions were abundant in soil environment (Mackelprang et al 2017, Nacke et al 2014).


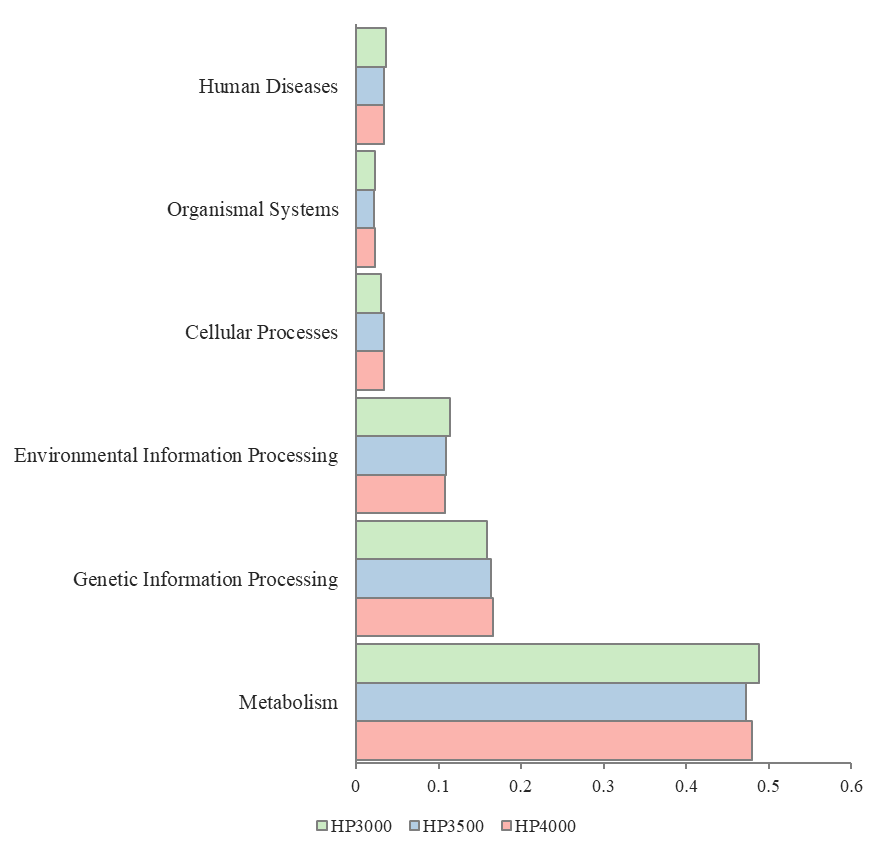


**A**


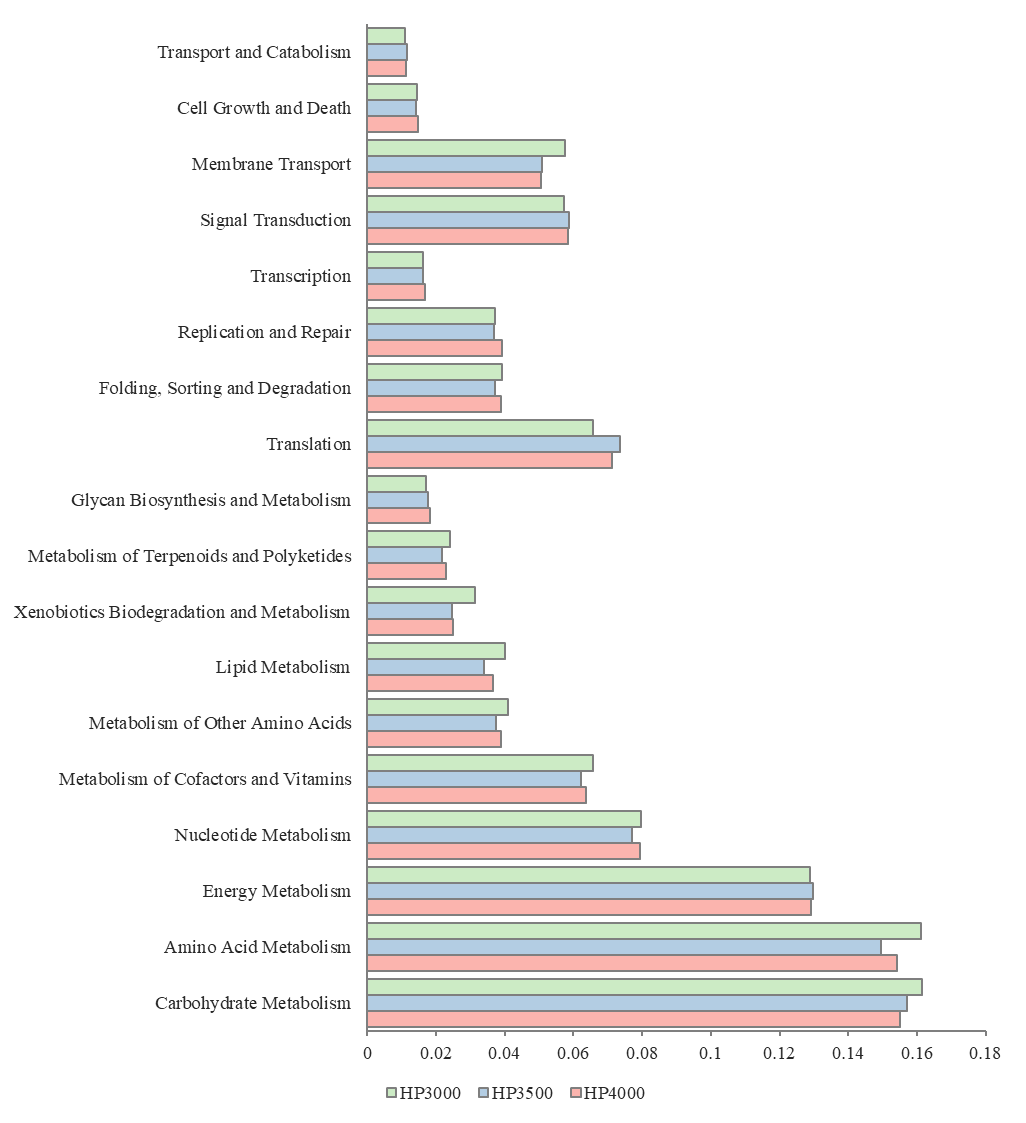


**B**


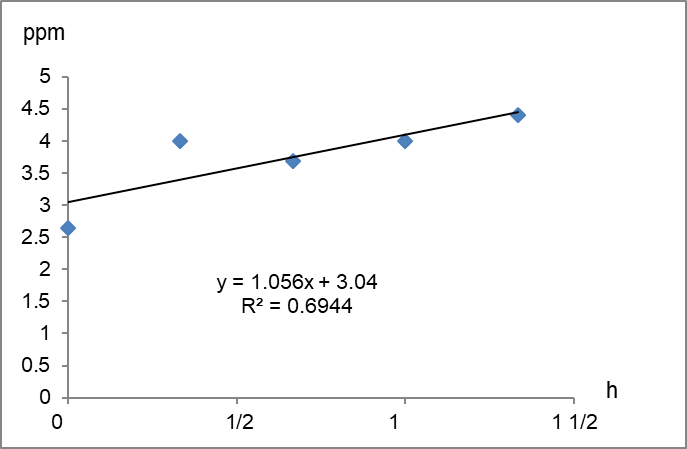


**D**


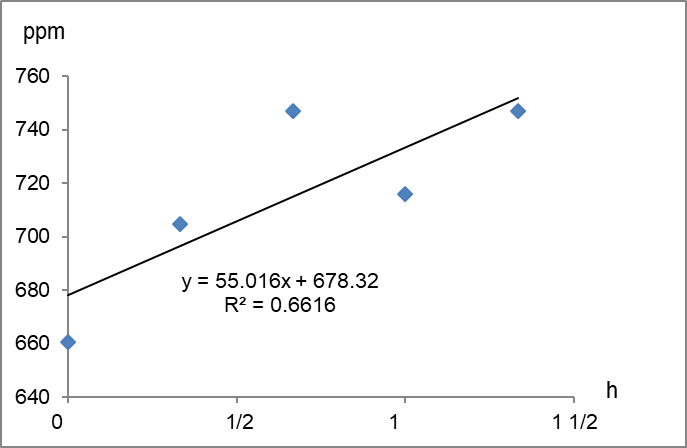


**C**


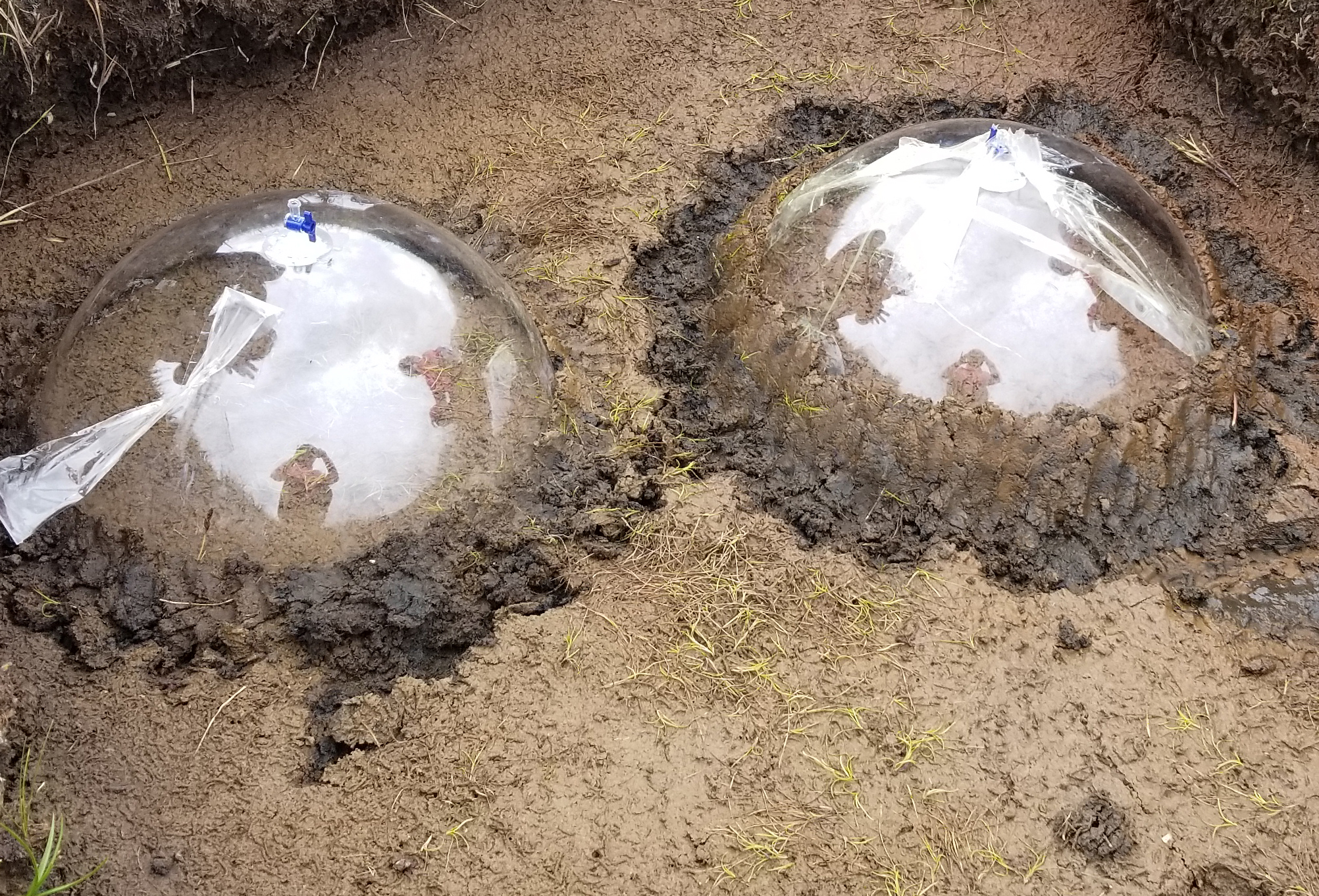


**A**


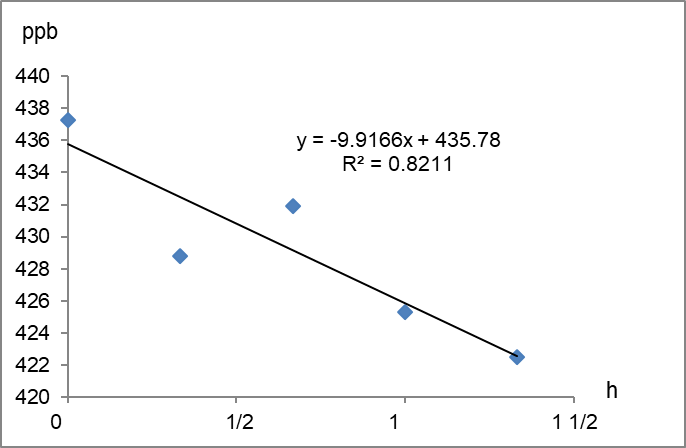


**B**

Figure S10. **A.** Picture of the on-site gas phase measurement. The relationships between time (h) and the gas concentration of N_2_O (**B**), CO_2_ (**C**) and CH_4_ (**D**) in the chamber in field. The slope value of the fitted curve was used to calculate the emission or consumption rate of gas in the chamber. In field, the temperature was 15 ℃ (288.15 K); the atmospheric pressure was 0.61 atm; the volume of the chamber was 7.5 L; the bottom area of the chamber was 0.102 m^2^.

Reference

Mackelprang R, Burkert A, Haw M, Mahendrarajah T, Conaway CH, Douglas TA *et al* (2017). Microbial survival strategies in ancient permafrost: insights from metagenomics. *Isme J* **11:** 2305-2318.

Nacke H, Fischer C, Thurmer A, Meinicke P, Daniel R (2014). Land Use Type Significantly Affects Microbial Gene Transcription in Soil. *Microb Ecol* **67:** 919-930.
